# Christmas Festivities

**Published:** 1905-12-23

**Authors:** 


### Christmas Festivities.

It is curious to observe the extent to which what may be called the "Charles Dickens Christmas," the period of much flesh eating, of much wine bibbing, and of not a little jovial intoxication, has been superseded in recent times by a festival in which we have surrendered ourselves into the hands of retail trade, and spend a great deal of money in giving more or less trumpery and useless presents to people who, in the majority of cases, do not either want them or know what to do with them. We read that this year the customary supply of Russian turkeys to the London market will not be forthcoming, and that, unless the deficiency be made good from other countries, the birds at our disposal will be some half million in number less than has of late been usual. They would, no doubt, be described by poulterers as "fine Norfolk," but we greatly doubt whether their absence will really be a matter for condolence. It will probably enable a certain proportion of home growers to obtain better prices for their goods, and it may perhaps serve to remind some of the would-be purchasers that there are, after all, better ways of spending money than in eating and drinking in excess of the requirements of the body.

Few things are more curious than the change which has come over public opinion in respect of eating customs, not merely in relation to Christmas, but equally with regard to the daily habits of our lives. It is not difficult, in an age of comparative enlightenment, to see that there is no manifest connection between the birth of the founder of our religion and the orgies with which that event was at one time commonly celebrated; nor is it difficult to refer the commencement of these orgies to a time when the clergy condemned industry on holy days, and left the uneducated faithful with no other resource than festivity. But the change which seems recently to have come over the observance of Christmas is only a variation of one that is much more general—that is to say, of a tendency towards moderation, in the use both of food and of drink, which would have induced our grandparents and great-grandparents to regard us as unmitigated milksops. The tendency to excess, where it yet lingers, is either the mere animal gorging of the labourer at his annual club-feast, or it is a consequence of the temptations offered to cultivated palates by the daily presence at table of exquisite cookery and of delicately flavoured wines. Even among those by whom these luxuries can be commanded we hear of influential seceders from customary practices, and the newspapers have lately published lists of distinguished people who are beginning to subsist chiefly upon nuts. These individuals may certainly expect to be spared any necessity for an annual resort to some German watering-place, even if they derive no other advantage from the peculiar form of abstemiousness which they have thought fit to adopt.

There can be no doubt that increased moderation in the consumption of food would be a practice of great national advantage; for we do not think there could be any disagreement, even among doctors, as to the amount of mischief that is done, and that is expressed in the form of diminished national longevity, by habitual excess even in a small degree. There is often a soul of good even in things evil, and no doubt excess in eating has been greatly increased and cultivated by the practice of using meals as convivial occasions, and by considering an abundant and varied supply of food and drink to be an indispensable evidence of hospitality and of kindly feeling. We are probably very far, either at Christmas or at any other time, from the wholesome practice of only eating to live; but it may be confidently hoped that the nation is in large measure emancipating itself from the opposite extreme of living only to eat. If it be true, as we think it is, that an increase of moderation is manifest even at our appointed seasons of festivity, we may surely hope that a more just measure of our actual wants is coming into view, and that we may look forward to a time when our people will be content with supplying them.

